# Polarization in multidisciplinary perspective

**DOI:** 10.1093/pnasnexus/pgae425

**Published:** 2024-10-15

**Authors:** Eugen Dimant, Erik O Kimbrough

**Affiliations:** Center for Social Norms and Behavioral Dynamics, University of Pennsylvania, Philadelphia, PA 19104, USA; Smith Institute for Political Economy and Philosophy, Chapman University, Orange, CA 92831, USA

**Keywords:** polarization, measurement, norms, social media, misinformation

## Abstract

This article provides an introduction to and overview of the articles in the *PNAS Nexus* Special Feature on Polarization and Trust.

## Introduction

Political polarization is a growing concern that disrupts societies worldwide. It is not merely a matter of differing opinions but a phenomenon that deeply influences our psychology, social dynamics, political and economic decision-making, and even the functioning of democratic institutions. A key aspect of polarization, particularly affective polarization, is the emotional divide it creates between groups, often reinforcing entrenched identities that resist compromise and understanding. This divide exacerbates existing societal tensions, including those related to racial disparities, economic inequalities, and partisan hostility (1–3). Understanding the complex nature of polarization—how it operates both at the level of the individual and the collective—is crucial for addressing its pernicious effects, and this special feature collects papers from multiple disciplinary perspectives that contribute to this goal. The papers included in this special feature in *PNAS Nexus* emerged from an interdisciplinary workshop (“Directions of Polarization, Social Norms, and Trust in Societies”) at MIT in 2023, which highlighted the importance of studying polarization through various analytical lenses.

Understanding polarization requires a multidisciplinary approach because it is shaped by a range of factors like policy disagreements, incentives, social identity, norms, emotions, and media consumption, which interact at both individual and collective levels (as explained by Kish Bar-On et al. in their Perspective (4)). The interplay between these elements suggests that polarization cannot be adequately understood through a single lens. Instead, it requires integrated insights from political science, psychology, sociology, and economics. For example, political scientists might examine the structural drivers of polarization, while psychologists explore the emotional and cognitive processes that sustain it.

Highlighting this interdisciplinarity, Enke’s Perspective (5) proposes a unification of moral psychology and political economy, suggesting that we cannot understand peoples’ views about policy without understanding their moral commitments and that the latter cannot easily be separated from the economic environment in which they were formed. This special feature is grounded in the belief that bringing diverse approaches together is necessary to refine our theories of polarization and to develop more effective interventions.

To achieve the latter goal, diverse insights from the social sciences then need to be communicated to policymakers, designers, and entrepreneurs. In that spirit, Pentland and Tsai’s Perspective (6) in this special feature draws on literature from across the social sciences documenting problematic consequences of polarization in online spaces and highlighting one approach that has shown promise in reducing the impact of polarization: deliberative democracy. The authors apply human-centric and evidence-based design insights to propose improvements to an existing online platform for deliberation. Their effort represents but one example of a large and growing set of possibilities.

## Measuring polarization

Two papers in the special feature focus on measurement issues. Polarization is a hot-button issue, but how prevalent is it in reality? Are people more polarized in some regions or states than in others? Holliday et al. offer a national perspective in (7), showing that affective polarization is widespread across the United States. Their analysis was based on a massive, nationally representative survey, revealing that affective polarization is relatively uniform across US states and primarily associated with individual-level characteristics rather than regional differences. These results point to the need for broad, psychologically informed interventions to address affective polarization across the entire country.

Recent work has improved the ability of social scientists to separately measure the individual and the social foundations of political belief systems and polarization (8). Measuring norms (i.e. beliefs about what others expect me to believe) and personal views separately allows researchers to understand the extent to which the former shape the latter (e.g. (9)). One challenge in studying polarization with existing methods is that they force respondents to report singular group-specific norms, potentially overlooking or underestimating the co-existence of plural norms within a group. To address this shortcoming, Panizza et al. introduce a new method for measuring the entire “normative landscape” as it is perceived by members of a particular reference group in (10). They show that groups of Americans recognize the polarization of beliefs in American society and are generally aware of what subgroups of both Republicans and Democrats expect one another to believe and to support. However, applying the same method within partisan groups reveals a surprising degree of within-group heterogeneity. That is, partisans do not necessarily see themselves as adhering to monolithic views. Such in-partisan pluralism suggests opportunities for compromise and highlights the importance of examining the internal dynamics of partisan groups.

## Psychological roots of polarization

Several papers in this issue explore the psychological roots of polarization, focusing on how perceptions and emotions drive partisan divides. A key element of affective polarization is negative affect towards people from the “other side.” This is a social emotion and thus, it is natural to ask how it is kindled and encouraged by (perceived) social pressure from other in-group members. In this vein, You and Lee in Ref. (11) demonstrate that perceptions of in-group norms—how strongly partisans believe their own group harbors negative feelings towards the out-group—are critical in driving affective polarization. As it turns out, partisans seem to perceive more normative pressure to hate the out-group than most individuals personally prefer. You and Lee (11) report three studies, including a pilot experiment, which showed that correcting exaggerated perceptions of in-group norms can significantly reduce support for partisan violence and lower affective polarization. Their work emphasizes the importance of targeting in-group norm perception in interventions, proposing that strategies focusing on correcting these beliefs may be a helpful complement to interventions that address out-group perceptions alone.

In a closely related study, Pradella (12) reports cross-sectional data showing that partisans also personally feel more empathetic toward out-group members than they expect other partisans to feel. As in You and Lee (11) an experimental manipulation that provides information about in-group members’ personal approval of out-group empathy successfully increases self-reported empathy. However, in a second treatment, when partisans are told that in-group members disapprove of out-group empathy, self-reported empathy also increases. This may suggest that polarization can be reduced not just by correcting misperceptions but also by simply encouraging partisans to think about the ways in which their views are shaped by social forces. This is an important hypothesis for future work.

At the same time, affective polarization is also a reactive emotion. People tend to dislike those who they believe dislike them, and Pradella (12) also finds that partisans have exaggerated beliefs about out-group members’ lack of empathy for them. Santos *et al.* (13) show that these exaggerated beliefs may have dangerous political consequences. In particular, they find that support for antidemocratic practices depends crucially on both their belief that out-partisans also support such practices and their degree of out-partisan empathy (which, as Pradella (12)) shows, also relates to how much empathy they think out-partisans have for them). Like You and Lee (11), they demonstrate the importance of correcting misperceptions, showing that encouraging empathy-driven curiosity can reduce political divides and strengthen democratic commitment. In particular, the authors experimentally demonstrate that shocking people’s curiosity about out-partisans’ beliefs by teaching them the benefits of cross-partisan empathy can reduce support for antidemocratic practices.

Given the prevalence and potential consequences of exaggerated beliefs, it is natural to ask how such beliefs might be formed in the first place.

## Social media and the formation of polarized beliefs

The special feature also includes two papers exploring the role of social media in shaping polarization. Lees et al. investigate the role of social media in shaping partisans’ perceptions of out-group animosity in Ref. (14). They report a study of how partisans’ beliefs that the opposing party hates them are influenced by social media use. Using panel data from the 2020 US presidential election, they found that while static social media activity did not predict animosity meta-perceptions, increases in political posting over time led to more accurate perceptions of the opposing party. Their findings suggest that dynamic changes in social media behavior, rather than static use, may play a crucial role in shaping and correcting inaccurate perceptions of political opponents.

If increasing political activity online leads one to acquire more information, it may also matter from whom one acquires that information. Zimmerman *et al.* (15) study how people choose which social media accounts to amplify. They analyze Twitter (now X) behavior and study the decision to retweet others’ posts. Though it is widely known that people exhibit homophily on social networks; that is, they preferentially interact with similar others, Zimmerman et al. (15) also find evidence of acrophily—people seem to be attracted to relatively extreme co-partisans. Extreme partisanship is associated with stronger affective polarization, and if such voices are more likely to be amplified on social networks, then it is perhaps no surprise that people overestimate both how much co-partisans expect them to hate the out-group and how much the out-group hates them.

## Combating misinformation

Given the prevalence of biased beliefs and the fact that social media may exacerbate them, it is important to ask what kinds of interventions can effectively counteract these tendencies. List et al. examine how critical thinking can reduce vulnerability to misinformation, a key driver of polarization in (16). Their online field experiment during the 2022 Colombian presidential election revealed that a de-biasing video enhanced critical thinking, making individuals more skeptical of misinformation. This study highlights the potential of using critical thinking interventions as a tool to combat misinformation, particularly in politically charged environments.

Radkani et al. (17) also embrace the view that cognitive factors are key in attempts to reduce misinformation. They develop a simulation model of “debunking” interventions, i.e. efforts by authorities to debunk false beliefs, and they attempt to explain the conditions under which such interventions will succeed or fail to reduce polarization in citizens’ beliefs. Crucially, debunking actions influence citizens’ beliefs about the authority’s motivation. The key condition for effective debunking is thus that the authority is perceived as independent.

## Behavioral consequences of polarization

Often interventions are designed to provide people with information about what others are doing in order to encourage them to join in. Rand and Yoeli (18) show that—in a polarized environment—the effectiveness of such interventions may depend on who those others are. Information about a descriptive norm necessarily comes from a reference group, e.g. co-partisans or out-partisans. They report three studies showing that providing information about the masking behavior of out-partisans actually “backfired”—in the sense that Biden supporters reported more willingness to wear a mask when told that Trump supporters were unlikely to do so. The same manipulation had the usual reinforcing impact on a different behavior that has no partisan connotation.

Behavior like that described in Rand and Yoeli (18) is often explained by theories in which partisans are motivated to take actions that reaffirm their identities and conform to the expectations of their group members. Supporting one’s group can mean taking actions that are against one’s interest. In this light, Robbett et al. (19) investigate peoples’ willingness to selfishly help themselves at the expense of others, and they show that they are less willing to harm co-partisans than out-partisans when their choices are observable. However, when given “plausible deniability” such that their choices are not observed by anyone, they treat everyone the same. This suggests that identity-based norms are often perceived by their adherents as costly.

Perhaps surprisingly, such costly political expression often extends outside the lab, as highlighted in a study of corporate activism by Braga et al. (20). They conduct a meta-analysis of 72 studies on corporate activism, revealing that it has a small but positive effect on outcomes like social media engagement and public attitudes, on average, with stronger responses among younger audiences and those aligned with the political leaning of the activism. The meta-analysis further indicates that corporate activism may have long-term implications for brand loyalty and public trust, particularly in highly polarized markets (20).

Together these papers suggest that the impact of polarization extends beyond the political realm into public health, interpersonal interactions, and markets.

## Conclusion

The papers in this special feature provide a multidimensional view of political polarization, highlighting its complex nature, the various factors that drive it, and its consequences for individuals and society. This collection of research offers a broader understanding of polarization than is typically found in single-discipline studies, emphasizing the interplay of cognition, perceptions, norms, social pressure, moral justifications, and incentives. The research highlights the importance of context-sensitive interventions, as their effectiveness can vary depending on specific social or political environments (for a discussion, see Ref. (21)). These contributions also illustrate why polarization is such a pressing concern, showing how it can undermine social cohesion, distort public discourse, and create barriers to cooperation—essential elements for the functioning of democratic institutions. By examining polarization through multiple lenses, this special feature not only enriches our understanding of the phenomenon but also opens new avenues for research and intervention, aiming to foster a more informed and resilient society.

## References

[1] Bakker BN, Lelkes Y. 2024. Putting the affect into affective polarisation. Cogn Emot. 38(4):418–436. 38847476 10.1080/02699931.2024.2362366 PMC11182229

[2] Dimant E . 2024. Hate trumps love: the impact of political polarization on social preferences. Manage Sci. 70(1):1–31.

[3] Iyengar S, Lelkes Y, Levendusky M, Malhotra N, Westwood SJ. 2019. The origins and consequences of affective polarization in the United States. Annu Rev Polit Sci. 22(1):129–146.

[4] Kish Bar-On K, Dimant E, Lelkes Y, Rand D. 2024. Unraveling polarization: insights into individual and collective dynamics. PNAS Nexus. 3(10):10.1093/pnasnexus/pgae426. PMC11475466 39411081

[5] Enke B . 2024. Morality and political economy from the vantage point of economics. PNAS Nexus. 3(10):10.1093/pnasnexus/pgae309. PMC11475465 39411084

[6] Pentland A, Tsai L. 2024. Toward building deliberative digital media: from subversion to consensus. PNAS Nexus. 3(10):10.1093/pnasnexus/pgae407. PMC11475399 39411091

[7] Holliday DE, Lelkes Y, Westwood SJ. 2024. Affective polarization is uniformly distributed across American states. PNAS Nexus. 3(10):10.1093/pnasnexus/pgae310. PMC11475403 39411088

[8] Converse PE . 2006. The nature of belief systems in mass publics (1964). Crit Rev. 18(1–3):1–74.

[9] Groenendyk E, Kimbrough EO, Pickup MA. 2023. How norms shape the nature of belief systems in mass publics. Am J Pol Sci. 67(3):623–638.

[10] Panizza F, Dimant E, Kimbrough EO, Vostroknutov A. 2024. Measuring norm pluralism and perceived polarization in U.S. politics. PNAS Nexus. 3(10):10.1093/pnasnexus/pgae413. PMC11475397 39411096

[11] You ZT, Lee SWS. 2024. Explanations of and interventions against affective polarization cannot afford to ignore the power of ingroup norm-perception. PNAS Nexus. 3(10):10.1093/pnasnexus/pgae286. PMC11475411 39411087

[12] Pradella L . 2024. Testing the social pressure hypothesis: does in-party social pressure reduce out-party empathy? PNAS Nexus. 3(10):10.1093/pnasnexus/pgae358. PMC11475405 39411089

[13] Santos LA, Voelkel JG, Willer R, Zaki J. 2024. Positive beliefs about cross-partisan empathy can strengthen Americans’ support for democracy. PNAS Nexus. 3(10):10.1093/pnasnexus/pgae394. PMC11475743 39411099

[14] Lees J, Cikara M, Druckman JN. 2024. Why partisans feel hated: distinct static and dynamic relationships with animosity meta-perceptions. PNAS Nexus. 3(10):10.1093/pnasnexus/pgae324. PMC11475464 39411086

[15] Zimmerman F , *et al*. 2024. Attraction to politically extreme users on social media. PNAS Nexus. 3(10):10.1093/pnasnexus/pgae395. PMC11475624 39411093

[16] List JA, Ramirez LM, Seither J, Unda J, Vallejo B. 2024. Critical thinking and misinformation vulnerability: experimental evidence from colombia. PNAS Nexus. 3(10):10.1093/pnasnexus/pgae368. PMC11475746 39411082

[17] Radkani S, Landau-Wells M, Saxe R. 2024. How rational inference about authority debunking can curtail, sustain or spread belief polarization. PNAS Nexus. 3(10):10.1093/pnasnexus/pgae393. PMC11475407 39411098

[18] Rand DG, Yoeli E. 2024. Descriptive norms can “backfire” in hyper-polarized contexts. PNAS Nexus. 3(10):10.1093/pnasnexus/pgae303. PMC11475620 39411094

[19] Robbett A, Walsh H, Matthews PH. 2024. Moral wiggle room and group favoritism among political partisans. PNAS Nexus. 3(10):10.1093/pnasnexus/pgae307. PMC11475467 39411083

[20] Braga L, Grinstein A, Tardini M, Perin M. 2024. Understanding reaction to corporate activism: the moderating role of polarization. PNAS Nexus. 3(10):10.1093/pnasnexus/pgae313. PMC11475398 39411100

[21] Bicchieri C, Dimant E. 2023. Norm-nudging: harnessing social expectations for behavior change. Working Paper.

